# Editorial: Reviews in thoracic oncology

**DOI:** 10.3389/fonc.2023.1209924

**Published:** 2023-05-11

**Authors:** Nikolaos I. Kanellakis

**Affiliations:** ^1^ Laboratory of Pleural Translational Research, Chinese Academy of Medical Sciences (CAMS) Oxford Institute, Nuffield Department of Medicine, University of Oxford, Oxford, United Kingdom; ^2^ Oxford Centre for Respiratory Medicine, Churchill Hospital, Oxford University Hospitals NHS Foundation Trust, Oxford, United Kingdom; ^3^ Oxford Biomedical Research Centre, National Institute for Health Research, Oxford, United Kingdom

**Keywords:** thoracic, cancer, oncology, lung cancer, malignant pleural effusion (MPE)

This is a Research Topic on thoracic oncology. Thoracic malignancy is a term used to describe any cancer presented in organs, glands, or structures within the thoracic cavity. Lung cancer is the second most frequent malignancy after breast cancer, accounting for 2.21 million cases annually, and the leading cause of cancer mortality worldwide (1.8 million deaths) for women and men combined (1). Men exhibit almost two times higher lung cancer incidence compared to women. The global patterns of lung cancer incidence and mortality are heterogeneous reflecting the stage of the tobacco epidemic. Lung cancer patients often present with pleural metastases. Pleural involvement signals advanced disease and poor expected prognosis (2).

Better understanding of the disease has advanced and improved lung cancer treatment and clinical management. Patients may receive chemotherapy, radiotherapy, targeted therapy, immunotherapy, and surgery. Chen et al., present the evolution of lung cancer treatments and landmark studies over the last two decades. Lung cancer patient phenotyping and targeted treatments have extended survival and reduced side effects. Brigatinib is an anaplastic lymphoma kinase (ALK) inhibitor and is administered as first-line treatment to ALK-positive metastatic non-small cell lung adenocarcinoma patients. Xing et al., systematically reviewed the efficacy and safety of Brigatinib. Tyrosine kinase inhibitors is another type of lung cancer targeted treatment. Currently, mutation status is determined by examining lung tumor tissue biopsies. Hu et al., discuss the advances of PET/CT in establishing EGFR mutation status in lung cancer and their clinical significance.

The introduction of immune checkpoint inhibitors (ICI) revolutionized cancer treatment and extended survival. However, not all patients respond to ICI therapy and benefit in terms of survival. It is still not clear which is the cohort of patients that would benefit the most. Mizuno et al., discuss the current status and future perspectives of PD-1/PD-L1 immune checkpoint blockade in lung cancer. ICIs may cause immune-related adverse events. Hao et al., present the pathogenesis, risk factors, and clinical presentation of immune checkpoint inhibitor-related pneumonitis. Non-small cell lung cancer patients with mutations on the MET pathway present poor clinical outcomes. The development of targeted tyrosine kinase inhibitors and bispecific antibodies for MET genetic alterations have benefited this cohort of patients. Michaels and Bestvina discuss the evolution and current state of MET selective therapy. Surgery remains the first-line treatment for early stage lung cancer patients with resectable tumors. The surgical methods have developed resulting to smaller surgical traumas, fewer complications, and quicker post-operational recovery periods. Fuzhi et al., outline the importance of evaluating pulmonary function in individuals who have undergone surgery for lung cancer, as well as the alterations in pulmonary function that occur after surgery. Additionally, they discuss strategies for effective rehabilitation of pulmonary function and factors that may affect the success of such rehabilitation.

Metastasis is a major factor that leads to mortality, and approximately 90% of cancer deaths are attributed to metastases. Malignant pleural effusion (MPE) is a common clinical problem for patients with lung cancer. The conduction of large-scale randomized clinical trials advanced diagnosis and clinical management. However, treatment focuses on symptom relief and control of fluid accumulation (Addala et al., 2022; Zhao et al., 2022). Parotid and gastric metastases for patients with lung cancer are rare and thus not very well studied. Wang et al., and Tang et al., present two reviews on primary lung cancer with parotid and gastric metastases.

Financial toxicity refers to the adverse impact of cancer treatment expenses on a patient’s quality of life. Lung cancer survivors often experience a rise in unemployment, psychological stress and a decrease in wages, indicating the persistent impact of financial toxicity. Boulanger et al., reviewed the connection between financial toxicity, quality of life, and survival in high value care.

Carcinogen derived thoracic cancers including lung and oesophageal are amongst the most frequently diagnosed malignancies worldwide. Despite advances like the introduction of ICIs the clinical management of these patients remains challenging. Endotyping of lung cancer patients in combination with targeted treatments has improved survival. Metastases are common and more studies are necessary to understand the underlying biology. Finally, the effect of cancer on the financial sustainability and stability of patients is an important factor we need to investigate and gather more data.

## Author contributions

The author confirms being the sole contributor of this work and has approved it for publication.
